# Perceived barriers to maternal healthcare utilization: insights from skilled birth attendants in six high-burden states of northern Nigeria

**DOI:** 10.1186/s12889-026-28357-2

**Published:** 2026-06-29

**Authors:** Hilda Ebinim, Ese Akpiroroh, Mary Ajayi, Usen-Obong Sabbath, Jabir Jibril, Precious Ehize, Saheed Isiaka, Deborah Kolawole, Sunday Nto, Chioma Unogu, Rauf Rauf, Sunday Atobatele, Sidney Sampson, Eugene Eugene, Hilary Okagbue

**Affiliations:** 1 Sydani Initiative for International Development, Sydani Group, Abuja, Nigeria; 2 Sydani Institute for Research and Innovation, Sydani Group, Abuja, Nigeria

**Keywords:** Skilled Birth Attendance (SBA), Antenatal Care (ANC), Facility delivery, Healthcare Delivery Assistants (HDA), Place of Last birth (PLB)

## Abstract

**Background:**

Maternal mortality remains a major public health concern globally despite significant progress over the past two decades. Nigeria is among the countries with the highest maternal mortality rates. Within the country, marked regional disparities persist, with northern Nigeria experiencing a significantly higher burden. These disparities are shaped by a complex interaction of sociocultural norms, household power relations, geographic constraints, and health system limitations that influence women’s access to skilled birth attendance and facility-based delivery. The study explored skilled birth attendants’ perceived barriers to the provision of maternal healthcare services that prevent pregnant women from seeking appropriate care in government-based facilities.

**Methods:**

This study employed a qualitative research design to explore skilled birth attendants’ perspectives on barriers preventing women from accessing maternal healthcare services. Twenty-four skilled birth attendants were purposively selected from primary healthcare facilities across six high-burden northern Nigerian states: Bauchi, Kaduna, Katsina, Kano, Jigawa, and Niger. Data were collected through in-depth interviews using a semi-structured and pretested interview guide. The Three Delay Model guided the study to examine factors affecting women’s decisions to seek care, their ability to reach healthcare facilities, and their experiences receiving care within facilities.

**Results:**

The findings revealed multiple barriers across the three phases of delay. At the household level, cultural expectations, generational beliefs, and patriarchal decision-making structures limited women’s autonomy to seek skilled care. Mothers-in-law and husbands often influenced or determined childbirth decisions, sometimes discouraging facility delivery. Structural barriers also affected women’s ability to reach health facilities, including long travel distances, poor road infrastructure, and limited transportation options. Even when women arrived at health facilities, systemic challenges such as shortages of skilled health workers, inadequate medical supplies, insufficient delivery beds, financial constraints, and weak referral systems contributed to delays in receiving appropriate care.

**Conclusion:**

In conclusion, maternal healthcare service utilization in northern Nigeria is shaped by interconnected sociocultural, economic, and health system factors that contribute to delays in seeking, reaching, and receiving care. Therefore, addressing maternal mortality in this context requires comprehensive strategies that strengthen health system capacity, improve transportation and referral systems, reduce financial barriers, and promote gender-equitable decision-making within households and communities. Such integrated efforts are essential for improving access to skilled birth attendance and advancing progress toward global maternal health targets.

**Supplementary Information:**

The online version contains supplementary material available at https://doi.org/10.1186/s12889-026-28357-2.

## Background

Although the global maternal mortality ratio has declined by 40% since 2000, it remains far from the 2030 target of 70% [1]. Low- and middle-income countries accounted for nearly 95% of maternal deaths [2], with sub-Saharan Africa accounting for almost 70% of maternal deaths [1]. In Nigeria, maternal mortality is a leading public health problem, with estimates ranging from 512 deaths per 100,000 live births in 2018 to over 800 deaths per 100,000 live births in 2019 [3], Kahansim and colleagues [4] found disparities in the rising trend of maternal mortality between the north and the south regions of Nigeria, particularly in areas [5], where Purdah (female solitude) is practiced. Specifically, the study reported that disparity is often influenced by the proportional utilization of skilled birth attendants in southern Nigeria, compared to their northern counterparts [6]. Maternal mortality is often an outcome of the interaction of a variety of factors that manifest in any of the three maternity phases, underscoring the complexity of preventing maternal deaths [7]. For instance, studies have established factors to include direct obstetric causes, including, but not limited to, hypertensive disorders [8], infections [3], and obstructed labour [9], and or underlying factors such as delay in seeking care [10].

The maternity phases - prenatal, perinatal, and postnatal - are critical to prevent maternal and child deaths, as complications can arise, necessitating skilled, timely, and quality care [11, 12]. While increased facility-based deliveries and skilled attendance are vital to reducing maternal mortality, this can only be successful if other structural conditions are established [13]. Despite recent reductions in maternal mortality, multilayered conditions at the micro, meso, and macro levels remain a barrier to optimal reductions in MMR across several sub-Saharan African (SSA) countries [14]. At the micro level, barriers that contribute to an optimal decrease in MMR include financial or economic constraints [15], geographic barriers of caregivers [2], poor knowledge of obstetric danger signs [16], fear of being treated poorly by health workers [17], and cultural beliefs [18]. The meso-level factors are women’s decision-making autonomy [19] and lack of family support [20]. The macro conditions influencing maternal mortality include the shortage and misdistribution of trained medical personnel [21], inadequate and poor-quality prenatal and maternity care services [22], lack of equipment [23], poor infrastructure [24], and a weak referral system [2]. Together, these factors contribute to delays in accessing and receiving care, highlighting the importance of addressing barriers to healthcare service utilization [25] and the conditions under which services are provided to those seeking care across the maternity phases.

Reducing maternal mortality is central to Goal 3.1 of the Sustainable Development Goals (SDGs), aimed at the goal of 70 deaths per 100,000 live births by 2030 [26]. Progress towards this has been uneven, especially in low-resource settings where improvements in service coverage do not translate into better maternal health outcomes [27]. Achieving SDG 3.1 requires attention not only on the caregivers’ part, but also the systemic and structural deficiencies towards receiving quality care [28, 29]. This study operationalizes caregivers as pregnant women who require maternal healthcare services, especially in the first phase of maternity. The concept of *caregiver* is adopted within a larger study context (this study is an offshoot), as many of the service seekers already have children.

Therefore, this study explored skilled birth attendants’ perceptions to understand (from a third-party perspective) and document barriers affecting pregnant women’s seeking appropriate care in primary healthcare facilities, using the *Three Delay Models*. Acquired information will offer insights into healthcare workers’ pragmatic understanding of pregnant women’s maternal health-seeking behaviour.

## Methods

This study is part of a larger study assessing women’s access to skilled birth attendants in Northern Nigeria. The study adopted a qualitative research approach to obtain information from twenty-four (24) skilled birth attendants (SBAs) selected purposively across six (6) states - Bauchi, Kaduna, Katsina, Kano, Jigawa, and Niger – based on the high maternal mortality burden across the selected states. A three-tier approach was used for the selection of the study location. At the same time, in-depth interviews were conducted to elicit relevant information from SBAs on their perception of the conditions affecting caregivers in seeking and accessing quality maternal care in Northern Nigeria through a semi-structured interview guide.

### Selected study areas and the primary healthcare system in Nigeria

The study was conducted in six northern states: Niger State, Jigawa State, Katsina State, Kano State, Kaduna State, and Bauchi State. Niger State is one of the states in the north-central region of the country. It is bordered to the east by Kaduna State and the Federal Capital Territory, to the north by Kebbi and Zamfara States, and to the south by Kogi and Kwara States. Its western border makes up part of the international border with the Benin Republic. It is the largest state in the country by land area, and it is divided into 25 local government areas (LGAs), and its capital is Minna.

Jigawa State is a state in the North-west region of Nigeria. It borders the Republic of Niger to the north, Yobe to the northeast, Bauchi state to the south, Kano to the southeast and Katsina to the northwest. Jigawa state has 27 LGAs, and its capital city is Dutse.

Katsina State is located in the northwestern geopolitical zone of Nigeria. To the west, it borders Zamfara State, to the east, it borders Kano and Jigawa States, and to the south, it borders Kaduna State. Its northern border forms part of the national border with the Republic of Niger. It is the third most populous state in Nigeria. The state has 34 local governments, and its capital city is Katsina.

Kano State is located in the northwestern region of Nigeria. It is the most populous state in the country. It borders Katsina state to the northwest, Jigawa state to the northeast, and Kaduna state to the southwest. The capital of Kano state is Kano, and it comprises 44 LGAs. Kaduna State is located in the Northwest zone of Nigeria, and its capital is Kaduna. It borders Zamfara and Katsina States to the north, Kano state to the north-east, Bauchi and Plateau states to the east, Nasarawa state, Niger state and Abuja to the south. It is the third-largest state by population. Kaduna state consists of 23 LGAs. Bauchi State is located in the northeastern region of Nigeria. Its capital is Bauchi. It is bordered by Jigawa state to the north and Yobe state to the north-east. Gombe state to the east, Taraba and Plateau states to the south, and Kaduna and Kano states to the northwest. Bauchi state has 20 LGAs.

Primary healthcare (PHC) was formally recognized as a fundamental approach to achieving access to essential health services following the 1978 Alma-Ata Declaration. The goal of PHCs is to ensure equitable, accessible and community-based care, emphasis on with emphasis on prevention, health promotion, disease prevention, treatment, and rehabilitation to improve health outcomes and reduce disparities, especially in underserved areas. PHCs serve as the first point of contact within the healthcare system, and they play a central role in the delivery of essential maternal and child health services [30].

### Study participants and selections

The study participants were skilled birth attendants providing care to women of reproductive age (15–49 years) residing in identified communities across two LGAs from each of the selected states. In the first tier, the LGAs were purposively selected based on the burden of maternal mortality and skilled birth attendants’ coverage. In the second tier, two LGAs - one urban and one rural LGA - were purposively selected in each state based on the population density of women of reproductive age and community-level engagement. The third tier involved purposively identifying skilled birth attendants (midwives and nurses) from selected Apex primary healthcare facilities in each of the selected LGAs, to ensure inclusion of relevant voices. The facilities were selected based on proximity, maternal health services, and patients’ inflow. The apex facilities in each LGA meet the three criteria. Four in-depth Interviews (IDI) were conducted (two per LGA) in each state, with each IDI being conducted with one midwife and/or a nurse in two apex primary health facilities within the selected LGAs.

### Analytical framework

**Fig. 1:**
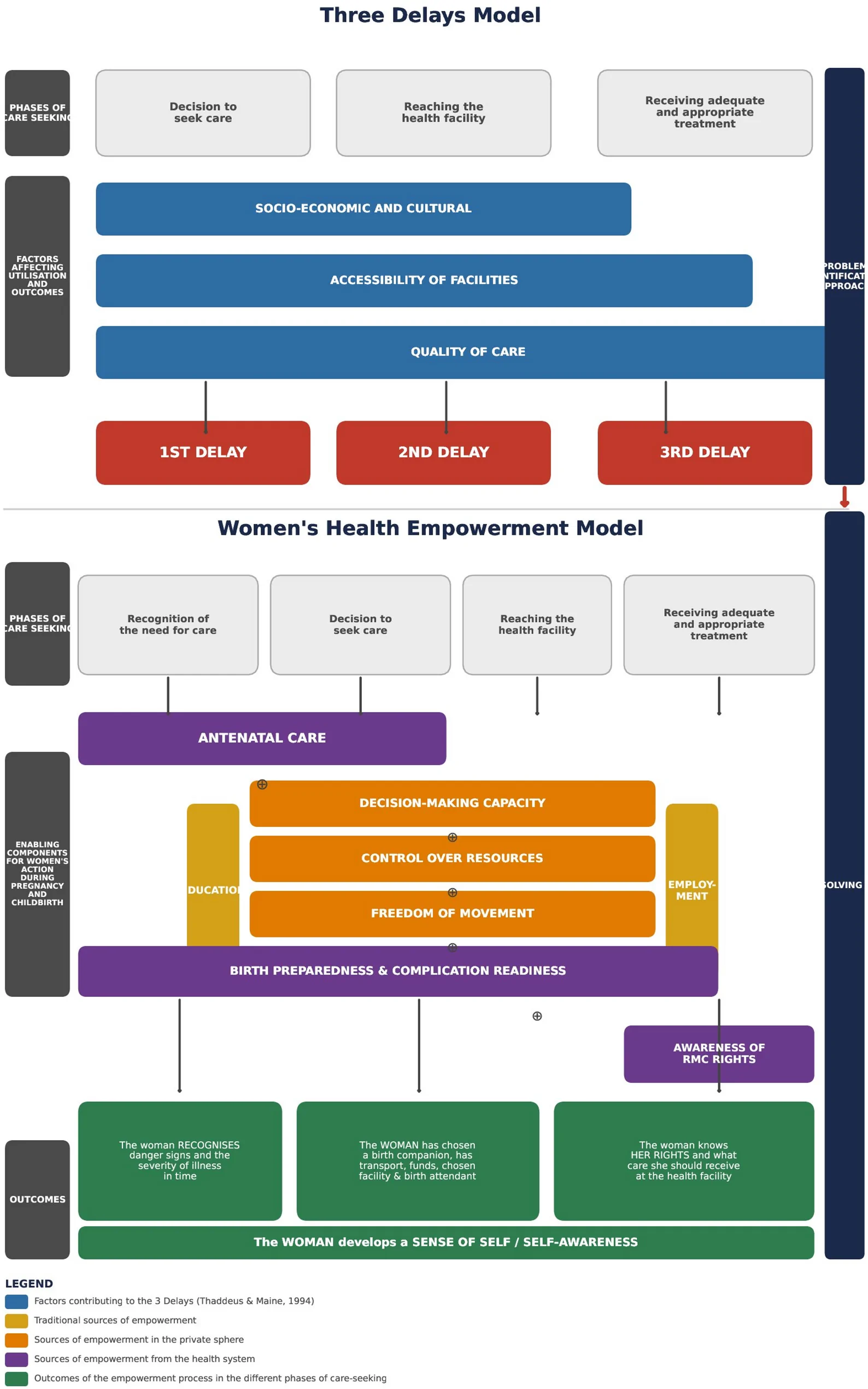
Women’s health empowerment model

The large study adopted the Women’s Health Empowerment Model (WHEM) as a theoretical framework guide (Fig. 1). However, this study specifically adapted the Three Delay Model from WHEM to systematically assess and document skilled birth attendants’ perceptions of the barriers affecting pregnant women’s seeking appropriate care in primary healthcare facilities. The diagrammatic representation of the study findings illustrates the adapted version of WHEM.

The model draws on insights from the Three Delays Model to promote autonomy, preparedness, and system responsiveness by shifting from an analytical perspective on delay in seeking maternal health to a women-centred approach. The Three Delay Model (3DM) was proposed in 1994 by Thaddeus and Maine to identify indirect factors that contribute to maternal death between the onset and outcome of an obstetric complication. The framework identifies the three critical phases that can prevent timely access to maternal care:

1. Delay in deciding to seek care.
2. Delay in reaching an adequate health facility.
3. Delay in receiving adequate care at the facility [31].

The WHEM cross-cut focus on women’s agency, preparedness, and a responsive health system. Furthermore, delays in decision-making, reaching care, and receiving care are shaped not only by individual actions but also by policies, systems, and the governance environment, which can enable or constrain access to skilled birth attendance.

### Data collection instrument

Semi-structured interview guides were developed and pilot-tested in the research team’s residential state to ensure clarity and cultural sensitivity. The in-depth interview guides explored skilled birth attendants’ perceptions of the conditions that affect pregnant women’s ability to receive maternal healthcare. The guide was adapted from the Women’s Health Empowerment Model (WHEM) framework, using the three delay models and aligned with the study objectives [31] to obtain information from skilled birth attendants at the facility level. Some of the questions include (i) Do women in this community usually deliver at this facility? (ii) From your experience, what are the main reasons some women come to give birth at this facility? Why do you think some women would not come to this facility to give birth? (iii) Have you observed instances where women could not access care due to the unavailability of staff?

### Data collection procedure

Twenty-four (24) in-depth Interviews (IDIs) were conducted across two LGAs from each of the selected states to obtain information from skilled birth attendants at selected primary healthcare facilities in those LGAs. This choice of selection was premised on the conditions that (i) it aligns with international standards, and (ii) it also limits repetition of information within the same locality. Participants were purposively selected based on their availability at the time of data collection (in some facilities, there were two nurses, while a few had one nurse and one midwife). Before the interview commenced, the study’s purpose and procedures were explained to participants, and verbal informed consent was obtained from all participants. The interviews were conducted mainly in the local language (Hausa), with a little English mixed in. All interviews were recorded on an audio device and labelled appropriately. The interviews lasted between 28 min and 43 min. A note-taker accompanied the interviewer to provide additional support by taking detailed notes to capture nonverbal cues and context that might not be captured in the recording. All recordings were uploaded by interview type and serial numbers. Interviews continued until thematic saturation was achieved, at which point no new themes emerged across successive interviews.

### Data analysis

After data collection, all audio recordings were translated verbatim and transcribed in Microsoft Word by experienced Hausa-speaking transcribers who were trained in qualitative research protocols. The research team collectively reviewed the transcript and deductively developed an initial codebook using the adapted WHEM framework. The transcripts were reviewed for accuracy and cultural nuance by another researcher outside of the team fluent in both languages. The transcripts were de-identified and coded in an alphanumeric format to indicate the states and LGAs where data were collected and to categorize the IDI sessions with the study participants. The states are listed numerically, while the LGAs are listed alphabetically. The transcripts were uploaded to Dedoose software (Version 14.0) for coding and analysis. The coding process also involved an inductive approach, where emerging codes not observed in the initial codebook were included in the analysis. Thematic and content analysis were employed. Findings were then synthesized to identify patterns, unique insights, and differences across states and respondents.

### Reflexivity

Many of the research team members have extensive experience conducting and implementing maternal health projects and offer significant insight into the subject matter. Additionally, some of the authors also have extensive experience in interpreting qualitative data and employing various interpretative approaches, including retroductive logic. In addition, the research team consisted of public health researchers with experience in maternal health programming. Regular reflexive discussions were conducted throughout coding and interpretation to minimize preconceived assumptions. Therefore, the study findings were interpreted and synthesized as objectively as possible. Although none of the researchers has lived in any of the communities where the study was conducted, we were constantly aware of avoiding any form of bias or comparison to our residential setting.

## Results

### Sociodemographic characteristics

This result shows the sociodemographic characteristics of the participants. A total of 24 SBAs were enrolled in the study, and more females (79.2%) participated in the study than males (20.8%). More than half (52%) of the participants were between the ages of 29–38% and just 4% of the participants were between the ages of 49–58%. 33.3% of the SBAs have a Bachelor of Scienc**e** (BSc) degree, while a much higher proportion (66.7%) have an Ordinary National Diploma (OND) degree. Of the 24 enrolled SBAs, 62.5% of them are nurses and 37.5% of them are Community Health Extension Workers (CHEW).

**Fig. 2:**
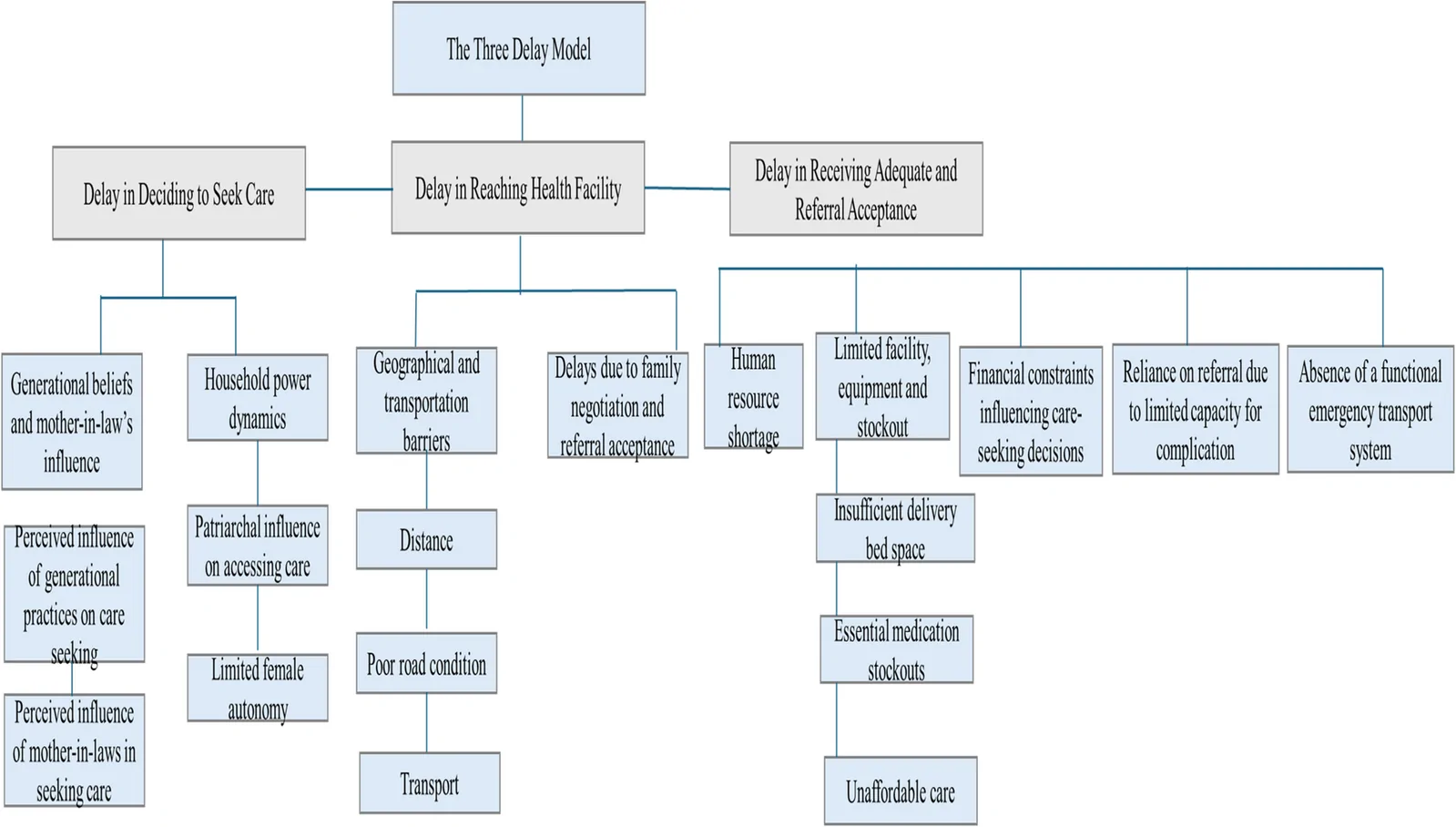
Thematic framework depicting skilled birth attendants’ perceptions of barriers to pregnant women receiving maternal healthcare in northern Nigeria using the Three Delay Model

The study’s findings are presented through a thematic analysis that identifies and describes skilled birth attendants’ perceptions of caregivers’ experiences in accessing care, using the Three Delay Model. The responses have been categorized into three themes: (i) Delay in decision to seek care, (ii) Delay in reaching a health facility, and (iii) Delay in receiving adequate and quality care. These themes have been recategorised into sub-themes (Fig. 2).

### Delay in deciding to seek care

The findings under this theme are considered the foundational elements that influence caregivers’ (pregnant women) decisions to seek care in a primary health facility. These are primarily premised on cultural and economic conditions in a household and/or family setting. To properly illustrate the healthcare workers’ perceptions, we have identified three sub-thematic positions under this theme.

#### Generational beliefs and mother-in-laws’ influence

Discussion with the study participants evoked conversations around the shared experiences of previous mothers (such as aunties, grandmothers, and other key female relatives) surrounding young women of reproductive age, their use of unskilled birth attendants, and the success associated with it. Participants were also keen to share that several mothers-in-law play key roles in preventing some pregnant women from using the health facilities, either as a result of their beliefs or personal experiences relating to the use of skilled birth attendants.

(i) Perceived influence of generational practices on care-seeking.

Interestingly, our study participants believed that generational practices within a family with several successful childbirth experiences without consulting skilled and qualified care in the health facilities remain a battle to be conquered in various communal settings. These experiences shared by relatives of current pregnant women, they believe, motivate their interest to follow a similar pathway for their maternal.

*“Like in some cultures*,* if someone’s kindred have never given birth in the hospital*,* then it becomes a norm*,* and they keep saying that they don’t give birth at the hospital in their family*,* so she cannot come to the hospital to give birth”* (SBA/IDI/1A/HEALTH FACILITY LEVEL/2025).

*“They just prefer to give birth at home because of what their family members are used to doing. They will say that their mothers before them did it*,* and it worked”* (SBA/IDI/6A/HEALTH FACILITY LEVEL /2025).

(ii) Perceived influence of mothers-in-law in seeking care.

Some of the study participants also highlighted that some pregnant women avoid the use of health facilities because their mothers-in-law are often convinced that their daughters-in-law should follow in their footsteps, even if it is against the caregiver’s wish to deliver her child at home.

*“Some of the mothers-in-law will not allow them to take them to the hospital; they will say that you must deliver at home”* (SBA/IDI/1D/HEALTH FACILITY LEVEL/2025).

*“Some people wish they could come*,* but their mothers-in-law* will not grant them permission” (SBA/IDI/1D/HEALTH FACILITY LEVEL/2025).

#### Household power dynamics

In this context, our study participants highlighted that deep-rooted household power imbalances influence care-seeking behaviours. Women’s access to care often depends on spousal approval and broader household hierarchies, which reinforce women’s subordinated position and their role in delaying access to care.

(i) Patriarchal influence on accessing care

According to study participants, spousal authority was also identified as a significant barrier to timely care-seeking among women. Participants noted that the need for husbands’ permission often delayed or prevented women from accessing maternal health services, as fear of disapproval or repercussions discouraged them from seeking care independently. Some participants particularly highlighted that scenarios such as this subsequently have chilling effects on neighbouring pregnant women.

*“For some of these women*,* their husbands have not given them permission to come”* (SBA/IDI/6E/HEALTH FACILITY LEVEL/2025).

*“Truly*,* it is one of the challenges that we do face*,* as the husband can also stop the woman from coming to the hospital. A woman can tell you that she come to the hospital to see someone at the hospital and then the family will start saying some kind of things to her and this can stop the other women from coming to the hospital too so they won’t tell or do the same thing to her”* (SBA/IDI/1A/HEALTH FACILITY LEVEL/2025).

(ii) Limited female autonomy

Further conversations with the study participants uncovered an additional level of thoughts, as some of these skilled birth attendants believed that women lack the agency required to make self-based decisions, even if such a decision is necessary to save their lives because they are confined to believing they need approval.

*“Many women do not believe they can make decisions by themselves. They believe they must accept permission from their in-laws or their husbands”* (SBA/IDI/4E/HEALTH FACILITY LEVEL/2025).

*“Some women are not given permission by their husbands or family members*,* so they stay at home*,* because they don’t think they can make the decision for themselves”* (SBA/IDI/6E/HEALTH FACILITY LEVEL/2025).

### Delay in reaching health facility

Following the decision to seek adequate care, pregnant women often face structural barriers beyond their control that may hinder access to timely care at an appropriate health facility. The respondents emphasized the difficulties that contributed to the delay in reaching the facilities. These difficulties include long distances to the facilities, rough terrains, and inadequate transportation services, making it difficult to access care.

#### Geographical and transportation barriers

Discussions with our participants revealed that the healthcare workers are aware that the distance between the health facility and some of the pregnant women’s communal settings remain a major impediment to accessing care, even when pregnant women desire it, and make an effort to access care. Participants also believed that the conditions of the road and limited forms of road transportation, especially from distant communities to the health facilities, further exacerbate the situation of distance.

(i) Distance

Some facilities are geographically inaccessible due to long distances, especially for women who reside in remote villages or far-to-reach settlements and are likely to face difficulty in successfully reaching the facility in a short time period. Participants believe that this is a major contributor to the limited number of pregnant women in health facilities.

*“Well*,* I think the reason some people don’t come to the hospital to give birth might be because of the distance of the hospital to their house”* (SBA/IDI/1B/HEALTH FACILITY LEVEL/2025).

*“Some people are from distant places; some can’t come from that distance”* (SBA/IDI/4D/HEALTH FACILITY LEVEL/2025).

(ii) Poor road condition

Our study participants also established that even when facilities are available, bad road conditions, rough terrain, and the lack of bridges can make prompt access to care difficult. During the rainy season, roads may be slippery and inaccessible due to flooding.

*“Some in this rainy season*,* the weather and road are not good*,* they cannot come out to deliver this here honestly*” (SBA/IDI/6G/HEALTH FACILITY LEVEL/2025).

*It is a barrier*,* we have a problem*,* when it’s raining season*, *for hard-to-reach areas*. (SBA/IDI/1D/HEALTH FACILITY LEVEL/2025).

(iii) Transport

Additionally, participants noted that women may experience delays in reaching the facility due to the unavailability of a functioning transportation service, especially in rural communities where there are few vehicles to convey them to health facilities when labour begins.

*“Some don’t have a means of transportation until they go out to the main road to get one. If they are not lucky*,* it will take them a lot of time before they get transport that will bring them down here”* (SBA/IDI/6G/HEALTH FACILITY LEVEL/2025).

*“Yes*,* transportation*,* like*,* those people living in the villages*,* they will say they don’t have somebody that will bring them to town”* (SBA/IDI/3A/HEALTH FACILITY LEVEL/2025).

#### Delays due to family negotiation and referral acceptance

Sometimes, after reaching a facility, delays occur because families are hesitant to accept referrals to higher-level facilities, due to perceived higher costs or denial of the severity of the condition. Health workers often spend significant time counselling families before referrals can proceed.

*“Sometimes the clients’ relations will not want the referral*,* you must counsel them*,* and after that*,* some will still not listen to you*,* and it is sometimes because of the purchasing power of the people. Because they know that if they go to a higher place*,* it may cost a lot. Sometimes they don’t even know; it is due to ignorance. They don’t even know that if they are being referred*,* it will make them get better care*,* but if you talk to them*,* they will understand*,* and they will later accept”* (SBA/IDI/2A/HEALTH FACILITY LEVEL/2025).

*“So sometimes*,* if we refer them*,* they will say*,* we can’t*,* we are not going. So*,* we used to encourage them on that”* (SBA/IDI/5B/HEALTH FACILITY LEVEL/2025).

### Delay in receiving adequate and appropriate care at the facility

On reaching the health facilities, the pregnant women are sometimes bedeviled with systemic challenges within the facility, such as a shortage of skilled birth attendants and inadequate equipment, that limit the quality of care offered to the pregnant women. These poses significant threat to the health of these women, especially in emergencies.

#### Human resource shortages

The healthcare workers who participated in our study shared that their reality as healthcare workers is very challenging, highlighting the severity of the situation of the human resource gaps within their system. Some of the participants specifically identify the significant difference between the Minimum Health Standard Package for Primary Health Care and the actual staffing reality in their facilities.

*“We have understaffing. The minimum health package requires 24 staff per facility*,* but here we just have 7. So*,* we cannot accommodate all the shifts; we only used to do morning and afternoon shifts*,* and night is on call”* (SBA/IDI/5A/HEALTH FACILITY LEVEL/2025).

*“Sometimes there’s a lack of staff*,* and then you have more clients or patients to care for. You must refer some of them to make sure everyone gets the attention they deserve”* (SBA/IDI/3E/HEALTH FACILITY LEVEL/2025).

#### Limited facility, equipment and stockouts

Another systemic barrier that the study participants discussed was the inadequacy of relevant equipment and commodities necessary for offering services to clients who visit the health facility. These inadequacies, they believe, limit their ability to provide adequate quality care to the clients, especially in cases of emergencies.

(i) Insufficient delivery bed spaces

Some of the study participants highlighted that the inadequate number of standard delivery bed spaces remains a major point of concern for many healthcare workers, especially in the cases of multiple requests for child delivery, as this often increases pregnant women’s wait time and delay in access to care.

*“We only have one proper delivery bed*,* and when more than one woman is in labour*,* we manage with another bed that’s not designed for delivery”* (SBA/IDI/6A/HEALTH FACILITY LEVEL/2025).

*“As you can see here*,* we have only two beds. Three women come to deliver*,* so they will not be delivered at the same time. So*,* when we take the one that is termed*,* her term has come*,* the delivery is timed*,* then we will quickly rush and screen her so when she gives birth*,* we take her to the resting room*,* clean the site*,* and bring another one”* (SBA/IDI/5B/HEALTH FACILITY LEVEL/2025).

(ii) Essential medication stockouts

Participants revealed that some facilities lack medications that are crucial for maternal care services, limiting their ability to provide appropriate care to women who come to their facilities for antenatal care and delivery

“*Honestly*,* we do not because as of now we don’t have Magnesium Sulphate to give those with pre-eclampsia as loading does*,* we don’t have. Those with pre-eclampsia must buy*” (SBA/IDI/4D/HEALTH FACILITY LEVEL/2025).

*Well*,* like I told you*,* it was around the lack of drugs and supplies in the labour unit* (SBA/IDI/5B/HEALTH FACILITY LEVEL/2025).

(iii) Unaffordable care

In this context, participants noted that the lack of free or subsidized services deters women from seeking skilled care when they need it, causing them to patronize cheaper and unsafe options.

*“Honestly*,* we do not always have free services. If we have it*,* we always give them for free*,* and you will always see them come. However*,* if they ask them to come and pay 1000 or 2000*,* you will see that they will not always come”* (SBA/IDI/4G/HEALTH FACILITY LEVEL/2025).

*“The advice of what will make women come to the facility is*,* let it be that they will have everything free*,* like this village women that is what will encourage them to come*,* for instant recently*,* when we had they had to pay like N500 to N700 naira*,* some took a long time before they returned because they don’t have that money”* (SBA/IDI/4G/HEALTH FACILITY LEVEL/2025).

#### Financial constraints influencing care-seeking decisions

Participants highlighted that low socioeconomic status significantly determines women’s decision to seek care from skilled birth attendants, especially in low-resource settings where women depend solely on their husbands’ income. Poverty and financial constraints in several households across many rural and a few urban communal settings across the country worsen the delay in decision-making, with some women prioritizing household needs over healthcare. Out-of-pocket purchase of drugs due to frequent stockouts negatively affects the utilization of health care services. Participants recounted that when pregnant women or their relatives are asked to purchase essential drugs for delivery, they may get dissatisfied or frustrated due to the additional cost incurred while buying these drugs at a higher price outside the facility.

*“Maybe for a patient who is coming to deliver. And then*,* you just must accept that some drugs are out of stock. And you write for them to go and get it. They will start shouting: ‘Why is it not available here?’ Why is it that when they come for delivery*,* we’re now sending them to get this and that? And so*,* some other things”* (SBA/IDI/3E/HEALTH FACILITY LEVEL/2025).

*“If we can get everything to be enough like drugs that they won’t have to use their money to buy drugs and injections*,* you will see them coming”* (SBA/IDI/1B/HEALTH FACILITY LEVEL/2025).

#### Reliance on referral due to limited capacity for complications

Several primary health facilities admitted their inability to manage obstetric complications, leading to frequent referrals. Even though referral protocols exist, the interplay of other factors may delay care.

*“Eclampsia is dangerous…it’s a complicated thing that we cannot attend to. We used to give unclear and refer to the general hospital”* (SBA/IDI/5A/HEALTH FACILITY LEVEL/2025).

*“I immediately wrote a referral for the woman*,* asking them to take her to the higher facility. But the woman who came with her and brought her to the facility did not take it seriously. I had to call one of my volunteers to go and look for the tricycle. to transport this woman to the hospital. When we did not get a tricycle*,* we had to even go and look for the woman’s brother*,* and the brother now came to me by myself. I enter inside the napep with the woman*,* and we took her to General Hospital”* (SBA/IDI/2D/HEALTH FACILITY LEVEL/2025).

#### Absence of functional emergency transport systems

The lack of ambulances and reliable referral transportation was consistently identified as a critical obstacle, particularly for obstetric emergencies. Facilities often depend on informal transport arrangements such as tricycles or motorcycles, which are unsafe or unavailable. Participants shared that some facilities do not have an ambulance. One participant recounted a near-fatal delay during a suspected uterine rupture due to transport challenges.

*“Yes*,* we don’t have ambulances. We liaise with NURTW whenever we have a referral; we have their contact*,* so they will come and transport our patient”* (SBA/IDI/5A/HEALTH FACILITY LEVEL/2025).

“*If we had an ambulance*,* it would have been very easy*,* you cannot carry a woman on a bike because the situation is for her to be in an ambulance*,* so that’s why we really need an ambulance*” (SBA/IDI/2D/HEALTH FACILITY LEVEL/2025).

## Discussion

This study provides insights into skilled birth attendants’ perceptions of the conditions influencing pregnant women’s utilization of maternal healthcare services in northern Nigeria. The premise of this study is based on the Three Delay Model, which provides a conceptual lens for understanding the perceived conditions/factors that limit pregnant women’s access to maternal health care in northern Nigeria. These contributing factors were classified into (1) Delay in deciding to seek care, (2) Delay in reaching the appropriate facility, and (3) Delay in receiving adequate care. The themes were further divided into subthemes, which will serve as the foundation of the discussion of the study’s findings.

This study identified several subthemes under the first delay (delay in seeking care), including generational beliefs and household power dynamics, which are prevalent in Northern Nigeria. Our study found that women’s preference for unskilled birth attendance results from the successful use of these services by older female relatives, making this a generational norm that women may be forced to participate in. This is consistent with another study, which found that family members’ experiences and perceptions influence a woman’s place of birth [32]. Furthermore, patriarchal supremacy is perceived to influence the decision to deliver in a health care facility. Findings from our study revealed that although women may desire to give birth in health facilities, their spouses and their mothers-in-law may think otherwise, denying them access to care due to perceived high cost or influence of communal norms that see home birth as a sign of courage and strength. Our findings extend previous evidence by demonstrating that family influence may either facilitate or hinder maternal healthcare utilization [33, 34].

Geographical barriers such as long distances, difficult terrain, poor transportation systems, and the absence of a functional emergency transport system, as well as delay in consenting due to familial reluctance to agree, all impede pregnant women’s ability to access and utilize maternal health services in time. Women often face delays in accessing appropriate care due to the distance from the health facility. This is worsened by a poor road infrastructure, unfavourable weather conditions, especially during the rainy season, and inadequate transportation services, especially in rural areas, where women must rely on local transportation during important circumstances such as labour, obstetric emergencies, and complications during delivery in the facility, which can negatively influence their desire to seek care. Our findings corroborate previous studies, which established that the physical distance of women to the health facility, as well as transportation costs and road access, influenced the decision to deliver at home [35–37]. Even when these women reach the facility and a referral is needed, health care workers often spend time convincing the woman’s spouse or family to consent to the referral. This reluctance stems from perceived increases in health care costs, the absence of ambulance services, or the lack of reliable means of transportation, further delaying access to care and worsening labour outcomes. This mirrors evidence from a study that found that women die as a result of delays from home or hospital referrals due to socioeconomic or geographic factors [4].

Health care services are not only influenced by the shortage of healthcare staff but also by inadequate essential equipment, financial hardship, medical supplies, and bed spaces, and reliance on referrals due to limited capacity to manage complications, which hamper access to care. Our study identified that some facilities face severe understaffing, particularly during the night shift, resulting in fewer skilled birth attendants on duty. This staffing gap increases the workload of available workers, leading to burnout and, consequently, suboptimal maternal care. This corroborates another study that found that patronage of local birth attendants is influenced by poorly staffed health facilities, resulting in longer waiting periods, insufficient attention to patients, and, consequently, inefficiencies in the facility, reducing the utilization of health services [23, 24]. The burnout of health workers due to increased workloads may cause negative behaviours in these women, consequently leading to the choice of using traditional birth attendants. This aligns with a study that reported that the poor use of the formal health system in a community in Kaduna state is due to the belief that care providers have a negative attitude, leading women to give birth at home or at a traditional health centre [10]. The delay is further exacerbated by low socioeconomic status, which results in prioritizing needs over seeking adequate care. We found that some women often prioritized household needs and feeding over seeking adequate healthcare services due to limited income and poverty. In instances where these women opt for facility birth, their husbands’ financial capacity determines their child’s place of birth. This agrees with previous studies that established that low income significantly contributes to inequality in accessing quality health services, especially among women [6]. The absence of essential equipment, such as beds and facility commodities (medications, among others), negatively impacts demand for and utilization of quality care. After overcoming other out-of-pocket expenses, causing delays, the cost of purchasing unavailable drugs, the dearth of bedspaces, and the unavailability of surgical equipment may further discourage women from utilizing quality health care. A study conducted in a northern Nigerian state emphasizes that the absence of clinics and major equipment hinders utilization of care among caregivers [38].

The findings from this study indicate that the systemic challenges observed result from the interplay among the three-delay model, which is deeply intertwined and collectively reinforcing. The first delay is often initiated by cultural norms and household power dynamics, which are exacerbated by generational beliefs and practices and patriarchal decision-making. These factors attenuate the second delay by influencing consent for referral, transportation choice, and willingness to travel long distances to health facilities. On reaching the facility, systemic health-service challenges, including staff shortages, inadequate infrastructure, and limited supplies, contribute to the third delay, weakening the benefits of facility-based delivery. This interaction across the three delays reveals that delays should not be addressed in isolation; they should be addressed holistically to improve the utilization of care in northern Nigeria.

### Study limitations

This study has some limitations. The study may have been influenced by social desirability bias, as it seeks to understand the factors impeding the utilization of care from the skilled birth attendant’s perspective, which may not reflect women’s personal motivations, constraints, or interpretations of care-seeking behaviour, resulting in a partial representation of the demand side. Secondly, our study did not assess the strength of maternal care services offered to the seekers of care to determine the quality of care. The study’s scope was limited to SBAs’ perceived barriers to access to the healthcare services provided. The study focused on six states in northern Nigeria, limiting its external validity to those states. Lastly, the study was conducted primarily in government-owned facilities; as a result, perceptions of SBAs working outside public facilities were not captured.

### Implications for practice

The findings highlight several practical considerations for improving maternal healthcare delivery in high-burden regions of Nigeria.

First, strengthening the capacity of primary healthcare facilities is essential. Many facilities reported critical shortages of skilled birth attendants, delivery beds, and essential medicines. Addressing these shortages through improved workforce deployment, continuous training, and adequate supply chain management would significantly enhance the quality of maternal care and reduce delays in receiving treatment.

Second, community-based maternal health education programmes should be expanded. Cultural norms, generational beliefs, and the influence of mothers-in-law were reported as important barriers to facility delivery. Engaging community leaders, religious leaders, husbands, and older female relatives in maternal health education can help reshape social norms that discourage facility-based childbirth.

Third, strengthening referral systems and emergency transportation, including mobile clinic service, is crucial to improving access to care. Many facilities rely on informal transport, such as motorcycles or tricycles, during obstetric emergencies. Establishing functional ambulance services a structured referral coordination, and mobile clinic systems at the local government level would reduce delays in reaching higher-level care by taking care to hard-to-reach areas.

### Policy implications

The findings suggest several policy actions that could support improvements in maternal health outcomes.

At the health system level, national and state governments should prioritize investment in primary healthcare infrastructure, particularly in high maternal mortality regions. This includes recruitment and equitable distribution of skilled birth attendants, provision of essential obstetric equipment, and reliable supply chains for life-saving medications.

Policies that reduce financial barriers to maternal healthcare are also necessary. Expanding free or subsidized maternal services, including antenatal care, delivery services, and emergency obstetric care, would reduce out-of-pocket expenditures that discourage women from seeking facility-based care.

Gender-responsive policies should also be strengthened. Programs that promote women’s autonomy in health decision-making, as well as male involvement in maternal health education, can help address household power dynamics that delay care seeking.

Additionally, government and development partners should collaborate with local transport providers like the Union of Road Transport Workers, particularly in rural areas, to improve the emergency transportation system for pregnant women. In addition, improved road networks can significantly reduce delays in reaching appropriate health facilities.

Overall, coordinated multisectoral policies that integrate health system strengthening, gender empowerment, and infrastructure development are essential for reducing maternal mortality in northern Nigeria.

## Conclusion

This study highlights interconnected sociocultural, economic, and health system barriers that limit access to maternal healthcare in six high-burden states in northern Nigeria. Cultural norms, household decision-making dynamics, long distances to facilities, weak transport and referral systems, staff shortages, and limited medical supplies collectively contribute to delays in seeking, reaching, and receiving care. Policy responses should prioritize strengthening primary healthcare systems, improving workforce distribution, ensuring reliable supplies, expanding financial protection for maternal services, and developing functional emergency transport systems. Future research should incorporate the perspectives of women, families, and communities to better understand care-seeking dynamics and inform context-specific interventions to improve maternal health outcomes. Reducing maternal mortality in northern Nigeria requires interventions that simultaneously address household decision-making, transportation and referral barriers, and weaknesses in primary healthcare systems. Addressing only one level of delay is unlikely to achieve substantial reductions in maternal mortality.

## Supplementary Information

Supplementary Material 1.

## Data Availability

The datasets used and/or analyzed during the current study are available from the corresponding author upon reasonable request. However, the interview guide is available as supplementary material.

## Abbreviations

ANC: Antenatal Care
CHEW: Community Health Extension Worker
EmONC: Emergency Obstetric and Newborn Care
HDA: Healthcare Delivery Assistants
IDI: In–Depth Interview
LGA: Local Government Area
LMICs: Low–and Middle–Income Countries
MMR: Maternal Mortality Ratio
NHREC: National Health Research Ethics Committee
PHC: Primary Health Care
PLB: Place of Last Birth
SBA: Skilled Birth Attendant
SDG: Sustainable Development Goal
SSA: Sub–Saharan Africa
TBAs: Traditional Birth Attendants
WHEM: Women’s Health Empowerment Model
3DM: Three Delay Model
