# Supplementary material for: Perceived barriers to maternal healthcare utilization: insights from skilled birth attendants in six high-burden states of northern Nigeria
Source: BMC Public Health. 2026 Jun 29;26:2590. doi: 10.1186/s12889-026-28357-2 (PMC13576299; doi:10.1186/s12889-026-28357-2)
Supplement: Supplementary file 1 [file 12889_2026_28357_MOESM1_ESM.docx]

**In-depth Interview Guide for Skilled Birth Attendants (Nurses/Midwives/CHEWS)**

**Section A: Respondent description**

- HCW unique identifier:
- Sex:
- Health facility:
- Job title:
- Highest educational qualification earned:

**Section B**

**1. Background and Professional Experience**

- Can you describe your role and responsibilities as [Job Title]?
- How long have you been a [Job Title]?
- How long have you worked in this facility as [Job Title]?
- Why did you want to become a [Job Title]?
- Describe the training you have received on maternal and child health services. *Probe: list the trainings you have received (e.g., Emergency Obstetric and Newborn Care, respectful care)?*

**2. Experience working in the health facility**

- Could you walk me through your typical day at the facility? *Probe: What are your main responsibilities, and roughly how much time do you spend at the facility each day?*
- Can you describe your experience providing ANC services at this facility? *Probe: what challenges have you encountered in delivering quality ANC services*
- From your experience, what prevents women from starting antenatal care early or attending regularly? *Probe: Do some women not realize they are pregnant? Are there issues related to transport or cost?*

**3. Observations on Delivery Care**

- Do women in this community usually deliver at this facility? *Probe: If no, what are the most common reasons why?*
- Are women referred to this facility due to complications arising from care provided by a traditional birth attendant (TBA)? *Probe: If yes, what are the major complications and how are these handled?*

**4. Barriers and Facilitators to Facility-Based Delivery**

- From your experience, what are the main reasons some women come to give birth at this facility? Why do you think some women would not come to this facility to give births? *Probe: What are the challenges that prevent women from using this facility for maternity care? (Probe: distance to health facility, transportation, family influence, staff attitude and waiting time etc.)*
- Are there any community or facility-based support systems to help women in getting to the facility during labor? *Probe: If yes, explain*
- Are there cultural, religious, or community factors that influence women’s decisions to give birth at home or use Traditional Birth Attendants (TBAs)? *Probe: Does the community support deliveries with skilled birth attendants at health facilities? If yes, how?*
- In your experience, which women are more likely to give birth at a health facility? *Probe: Are there differences based on age, level of education, marital status, socioeconomic status, or distance to the facility? Are younger or first-time mothers more likely to use skilled birth attendants compared to older women?)*
- Are you able to accommodate every woman that wishes to give birth at the facility? *Probe: facility is underused, too little staff, not enough beds etc.*

**5. Relationships and Collaborations**

- Are there any existing collaborations or referral arrangements between this health facility and community health workers or traditional birth attendants (TBAs)? If so, how does this work?
- Are there efforts (past and ongoing) to engage men, family members, or community leaders in promoting facility-based delivery? If yes, please explain *Probe: Were the past efforts effective? If yes, why did they stop?*

**6. Staff Availability and Manpower**

- How many permanent staff are responsible for delivery services in this facility?
- Can you discuss any challenges this facility faces in regard to availability of staff. Probe: How does this impact delivery services?
- Have you observed instances where women could not access care due to the unavailability of staff? *Please explain and give examples.*
- Could you please describe the operational hours of this health facility? *Probe: What are the opening and closing times? Do you operate in shifts such as morning and night shifts?)*
- In your opinion, does the number of health care workers handling delivery services in this facility affect the quality of care women receive during deliveries? *If yes, explain how*

**7. Quality of Care and Facility Environment**

- How would you describe the availability of medicines, supplies, and equipment needed for safe delivery? *Probe: Have there been times when medicines or supplies were unavailable in this facility during delivery? Can you describe any situations where you couldn’t provide the needed care because of stockout of drugs or supplies?*
- What changes have you seen or what suggestions do you have to improve delivery services and patient satisfaction in this facility?

**8. Emergency Preparedness and Referral**

- What barriers affect timely referrals or transfers during emergencies*? (Are there challenges related to transportation, availability of ambulances, costs or communication between facilities)*
- What procedures are followed in this facility for dealing with emergencies or complications during labor? *In a place where there is a complication you cannot deal with here, what will you do?*
- Can you tell me about the follow up care you would provide for women after giving birth? (postnatal care, vaccination, where does this take place)
- Do you feel that the resources and capacity available at this facility are sufficient to cope with any complications that arise during pregnancy, labor and immediately after birth? *If no, what can be done to improve this?*

**9. Recommendations and Solutions**

- In your opinion, what could be done to improve women’s access and use of skilled birth attendants in this community?
- Do you think in general that the care women receive in rural areas during pregnancy and birth has improved over time or do you think there has been little change, for example in the last three years? *If yes, why*
- Do you feel that working in the health facility you have contributed to improvements in women’s health? Can you tell me in what way? [*if not, reasons why not. If yes, provide examples of how*]
- Can you tell me about anything you would like to see improved [*staffing levels/experience, resources, training opportunities, support, better publicity in the community*]
- Is there anything else you want to share about your experience or challenges as a skilled birth attendant?

Thank you for your time.
